# Comparative Evaluation of Esthetic Outcomes Among Different Dental Implant Systems in the Maxillary Anterior Region

**DOI:** 10.7759/cureus.112684

**Published:** 2026-07-14

**Authors:** Divya Sree Gunnam, Akshat A Chokshi, Srinu Kashana, Debadrita Ghosh, Muthuraj H. L., Jay Gohil

**Affiliations:** 1 Department of Public Health, University of New Haven, West Haven, USA; 2 Department of Dentistry, Taunton Village Dental, Oshawa, CAN; 3 Department of Pedodontics and Preventive Dentistry, Family Dental Clinic, Kolkata, IND; 4 Department of Dentistry, Global Smiles Dental, Indianapolis, USA; 5 Department of Prosthodontics, Crown and Bridge, Farooqia Dental College and Hospital, Mysuru, IND; 6 Department of Prosthodontics, Crown and Bridge, K. M. Shah Dental College and Hospital, Sumandeep Vidyapeeth (Deemed to be University), Vadodara, IND

**Keywords:** dental implants, esthetics, maxillary anterior region, pink esthetic score, prosthodontics

## Abstract

Introduction

Achieving optimal esthetic outcomes in implant-supported restorations in the maxillary anterior region remains a major challenge in implant dentistry. Implant design, implant-abutment connection, prosthetic rehabilitation, and peri-implant soft tissue stability may significantly influence the final esthetic outcomes. A comparative evaluation of different implant systems using standardized esthetic indices may assist clinicians in selecting appropriate implant systems for esthetically demanding cases. Therefore, this study aimed to evaluate and compare the esthetic outcomes of Straumann, Nobel Biocare, and Osstem implant systems in the maxillary anterior region.

Materials and methods

This prospective observational study was conducted in the Department of Prosthodontics, Crown and Bridge, over a period of two years. A total of 90 patients receiving single implant-supported restorations in the maxillary anterior region were enrolled and prospectively followed. Based on the implant system used, participants were categorized into three groups: Straumann, Nobel Biocare, and Osstem (n = 30 each). Esthetic outcomes were evaluated clinically using the Pink Esthetic Score (PES), White Esthetic Score (WES), and Visual Analog Scale (VAS) six months after definitive prosthetic loading. Statistical analysis was performed using one-way analysis of variance (ANOVA) for continuous variables and the chi-square test for categorical variables. A p-value < 0.05 was considered statistically significant.

Results

Among the study participants, 43 (47.8%) belonged to the 36-50 years age group, 56 (62.2%) were females, and 70 (77.8%) were non-smokers. Straumann implants demonstrated the highest mean PES score (12.1 ± 1.4), followed by Nobel Biocare (11.8 ± 1.5) and Osstem implants (9.9 ± 1.7), with statistically significant differences among the groups (p < 0.001). Similarly, the mean WES scores were significantly higher for Straumann (8.5 ± 0.9) and Nobel Biocare implants (8.3 ± 1.0) than for Osstem implants (7.1 ± 1.2) (p < 0.001). The mean VAS score was also the highest for Straumann implants (88.4 ± 7.2), followed by Nobel Biocare (85.6 ± 8.1) and Osstem implants (74.3 ± 10.5) (p < 0.001). Straumann and Nobel Biocare implants exhibited significantly superior esthetic outcomes than Osstem implants.

Conclusion

Within the limitations of the present study, Straumann and Nobel Biocare implant systems demonstrated superior esthetic outcomes compared with Osstem implants in the maxillary anterior region. The implant-abutment connection design, use of custom abutments, and prosthetic characteristics may significantly influence peri-implant esthetics and overall patient satisfaction.

## Introduction

With increasing esthetic awareness and functional demands, dental implant therapy has become a preferred treatment modality for the replacement of missing teeth, particularly in the anterior maxillary region [1]. Patient-related behavioral factors, including perceived benefits, esthetic expectations, and treatment acceptance, also play an important role in the success and acceptance of implant therapy [2]. In the anterior maxillary region, implant-supported restorations are expected not only to restore function but also to achieve superior esthetic outcomes because of the high visibility of this region during speech and smiling [3]. Achieving optimal peri-implant soft tissue contours, harmonious gingival architecture, and natural-looking prosthetic restorations remains a major challenge in implant dentistry [4]. Several factors, such as implant design, implant-abutment connection, abutment material, crown material, and surgical and prosthetic protocols, may significantly influence the final esthetic result [5].

Various implant systems have been introduced with modifications in macrodesign and microdesign to improve osseointegration, peri-implant tissue stability, and esthetic performance [6]. Parvini et al. [7] evaluated the esthetic and clinical outcomes following immediate implant placement and restoration and reported favorable peri-implant soft tissue stability and esthetic outcomes with both implant systems. Their findings emphasized the importance of implant design, prosthetic management, and soft tissue preservation in achieving predictable esthetic success in the anterior maxilla. Comparative evaluation of different implant systems using these validated parameters may help clinicians select the most appropriate implant system for esthetically demanding cases. The present study was conducted to compare the esthetic outcomes associated with different dental implant systems placed in the maxillary anterior region. The objectives of the study were to describe the demographic and clinical characteristics of the study population, compare implant- and prosthetic-related characteristics among the three implant system groups, and evaluate the association between implant system groups and esthetic outcomes, as assessed using the Pink Esthetic Score (PES), White Esthetic Score (WES), and Visual Analog Scale (VAS), under routine clinical practice.

## Materials and methods

Study design and setting

The present prospective observational study was conducted in the Department of Prosthodontics, Crown and Bridge, K. M. Shah Dental College and Hospital, Sumandeep Vidyapeeth (Deemed to be University), Vadodara, Gujarat, India. The study was conducted over a period of two years, from April 27, 2018 to March 30, 2020. The study protocol was reviewed and approved by the institutional ethics committee (SVIEC/DU/2018/Dent/april/25, dated April 14, 2018). This study was conducted in accordance with the ethical principles outlined in the Declaration of Helsinki [8]. Written informed consent was obtained from all patients before enrolment and treatment. 

**Figure 1:**
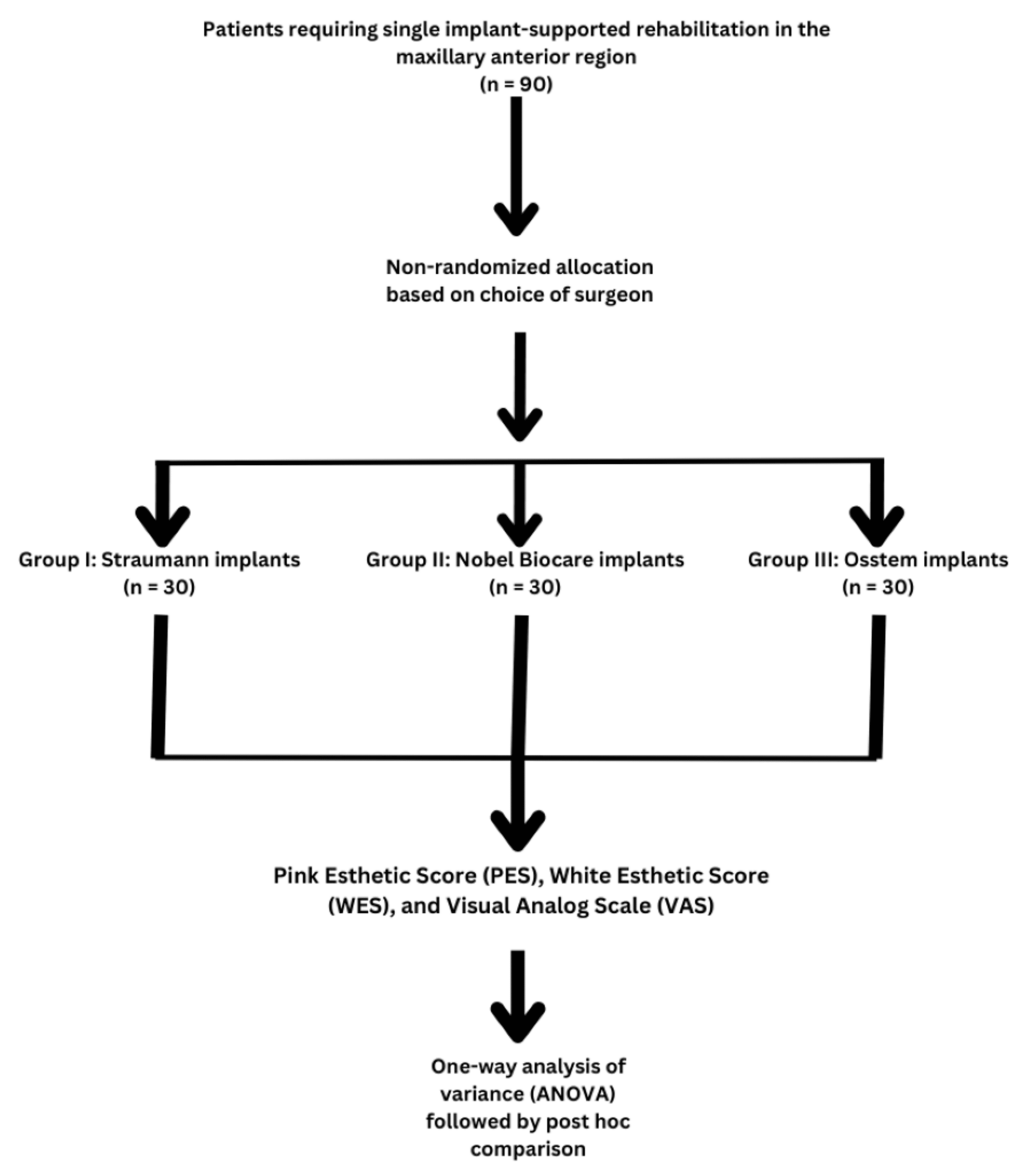
Study flowchart. Figure created by the authors using Canva (Canva Pty Ltd., Sydney, NSW, Australia). No artificial intelligence-generated content was used in the preparation of this figure.

Patients requiring single implant-supported rehabilitation in the maxillary anterior region were consecutively recruited and followed prospectively (Figure 1). Implant placement, selection of implant system, surgical protocol, and prosthetic rehabilitation were performed according to routine clinical requirements and the treating clinician's judgment. No intervention was made by the investigators regarding treatment allocation. Patients were followed through implant placement, osseointegration, second-stage surgery, and prosthetic rehabilitation. Esthetic outcomes were assessed six months after definitive prosthetic loading.

Sample size estimation

Sample size estimation was performed using the G*Power software version 3.1.9.7 (Heinrich Heine University Düsseldorf, Düsseldorf, Germany). The calculation was based on an anticipated medium effect size (f = 0.30) from a reference study by Barwacz et al. [9], study power of 80% (β = 0.20), and significance level (α = 0.05). The minimum required sample size was calculated as 84 dental implants. To compensate for possible exclusions and improve the precision of the analysis, 90 dental implants were included in the study, with 30 implants in each implant system group.

Study population and eligibility criteria

Patients requiring single implant-supported rehabilitation in the maxillary anterior region, extending from the right canine to the left canine, were recruited for the study. A total of 90 implants placed in 90 patients were included and categorized into three groups according to the implant system used, with 30 patients in each group. Implant selection was based on routine clinical decision-making and was not determined by the investigators. Patients aged 18-65 years requiring single implant-supported restoration in the maxillary anterior region (right canine to left canine) were included in the study. Patients with adequate bone volume for implant placement, satisfactory oral hygiene maintenance, and willingness to attend scheduled follow-up visits were considered eligible. Patients with uncontrolled systemic diseases, active periodontal disease, parafunctional habits (such as bruxism), a history of radiotherapy in the head and neck region, inadequate bone volume requiring extensive reconstructive procedures, poor oral hygiene compliance, failed osseointegration, peri-implantitis during the observation period, or incomplete follow-up were excluded from the study.

Implant systems evaluated

Group I included Straumann implants (Straumann Holding AG; Basel, Switzerland) with sandblasted, large-grit, acid-etched (SLA) surface technology and predominantly internal implant-abutment connection. Group II included Nobel Biocare implants (Nobel Biocare Services AG; Zürich, Switzerland) featuring moderately rough surface-treated implants with internal connections. Group III included Osstem implants (Osstem Implant Co. Ltd., Seoul, South Korea) with surface-treated titanium implants and internal/external connection configurations, according to the implant system used.

Preoperative assessment

All patients underwent comprehensive clinical and radiographic examinations before implant placement. Diagnostic impressions, intraoral examinations, and radiographic evaluations using intraoral periapical and panoramic radiographs were performed to assess bone availability, implant site conditions, occlusion, and esthetic requirements. The gingival biotype and smile line were clinically evaluated during treatment planning.

Implant placement procedure

Implant placement procedures were performed under local anesthesia using standardized aseptic surgical protocols by experienced prosthodontists and implantologists (AAC, DC). Osteotomy preparation was performed sequentially according to the drilling protocol recommended by the implant manufacturers. Implant dimensions, including diameter and length, were selected based on the available bone volume and prosthetic requirements.

Implants were placed either immediately following tooth extraction or in healed/early healed extraction sites in the maxillary anterior region, depending on the clinical condition and bone availability. In cases of immediate implant placement, atraumatic tooth extraction was performed with maximum preservation of the surrounding alveolar bone and soft tissues. Implant placement was performed to achieve optimal three-dimensional positioning and primary stability. Primary implant stability was achieved in all cases during implant insertion. The implant-abutment connection type, implant dimensions, placement timing, and loading protocol were recorded for all cases. Healing abutments or cover screws were placed according to the treatment protocols. Immediate provisionalization was performed in selected cases with adequate primary stability and favorable occlusal conditions, whereas delayed loading protocols were followed in the remaining cases. Patients received postoperative instructions and medications and were recalled periodically for follow-up.

Prosthetic rehabilitation procedure

Following an adequate osseointegration period, second-stage surgery was performed where indicated, and prosthetic rehabilitation procedures were initiated. Prosthetic impressions were obtained using standardized techniques. Abutment selection was performed according to the implant angulation, soft tissue profile, and esthetic requirements. Both custom and prefabricated abutments were used depending on the clinical condition.

Definitive prosthetic restorations primarily included all-ceramic zirconia crowns because of their superior esthetic properties, favorable translucency, and soft tissue compatibility in the anterior maxillary region. Lithium disilicate (IPS E.max) and porcelain-fused-to-metal restorations were used according to the clinical and esthetic requirements [10]. Cement- and screw-retained prostheses were fabricated according to the prosthetic indications. Occlusal adjustments were performed to establish functional harmony and minimize the occlusal overload. The final prostheses were delivered after verifying esthetics, marginal adaptation, occlusion, and patient satisfaction.

Esthetic evaluation

**Figure 2:**
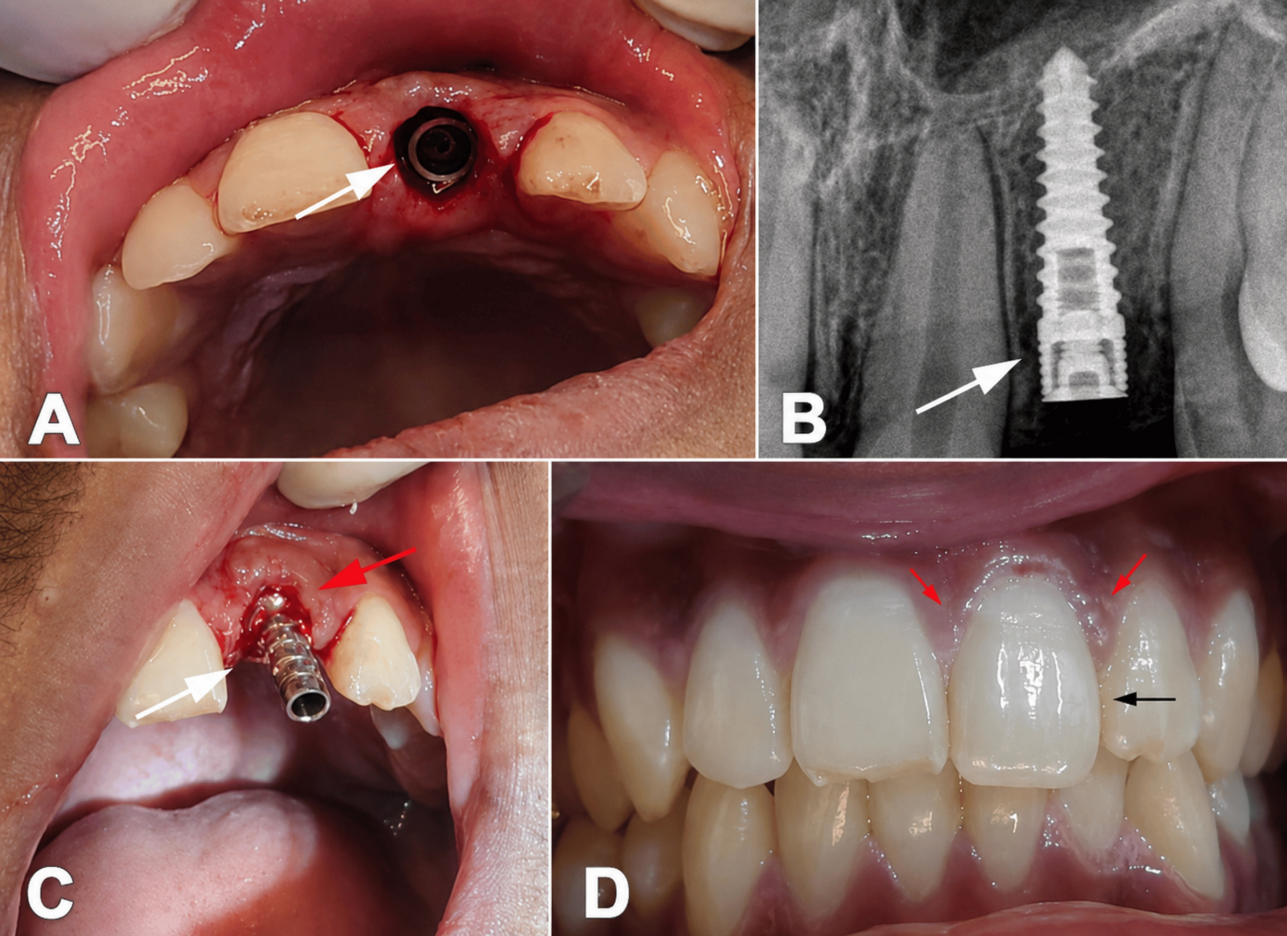
(A) Clinical view after implant placement; white arrow indicates the implant platform at the surgical site. (B) Immediate postoperative periapical radiograph; white arrow indicates the coronal portion of the dental implant. (C) Healing phase after implant placement; white arrow indicates the healing abutment, red arrow indicates the gingival margin. (D) Final implant-supported restoration; red arrows indicate the gingival margin, black arrow indicates the implant crown. Original images of the patient from the study, used with the patient's consent.

The esthetic outcomes of implant-supported restorations were evaluated six months after definitive prosthetic loading under standardized clinical conditions by two independent prosthodontists (MHL, JG). Esthetic assessment was performed using the PES, WES, and VAS [11-13]. The PES was used to assess peri-implant soft tissue parameters, including the mesial papilla, distal papilla, level and contour of the facial mucosa, alveolar process deficiency, and soft tissue color and texture. The WES was used to evaluate prosthetic characteristics, including tooth form, contour, color, translucency, characterization, and surface texture. Overall esthetic satisfaction was assessed using a VAS ranging from 0 to 100. All clinical examinations were performed under standardized illumination and patient positioning conditions to ensure consistency and reproducibility of the assessments (Figure 2).

Examiner calibration and reliability assessment

Before the commencement of the study, two experienced prosthodontists (MHL and JG) were calibrated for PES, WES, and VAS assessment using clinical cases not included in the study sample. Calibration sessions were conducted to standardize the interpretation and scoring criteria and minimize examiner variability. To determine intra- and inter-examiner reliability, 15 randomly selected implant cases were re-evaluated after a two-week interval under identical clinical conditions. Reliability analysis was performed using the intraclass correlation coefficient (ICC) and Cohen’s kappa statistics. An ICC value greater than 0.80 was considered to indicate excellent agreement. The obtained values demonstrated good to excellent reproducibility and consistency of the esthetic assessment parameters.

Data collection

Demographic and clinical variables, including age, sex, smoking status, gingival biotype, smile line, implant site, implant dimensions, implant-abutment connection type, placement timing, loading protocol, crown material, crown retention type, abutment type, abutment material, PES, WES, and VAS scores, were collected and systematically recorded.

Statistical analysis

Statistical analyses were performed using IBM SPSS Statistics for Windows, version 25.0 (released 2017; IBM Corp., Armonk, New York, United States) by a statistician who was provided with coded data (SK). Descriptive statistics were calculated and presented as frequencies, percentages, means, and standard deviations. The normality of continuous variables was assessed using the Shapiro-Wilk test. Variables with a normal distribution were analyzed using one-way analysis of variance (ANOVA), while categorical variables were compared using the chi-square test. Post-hoc comparisons were performed where applicable. Esthetic outcome measures, including PES, WES, and VAS scores, were compared among the three implant systems using one-way ANOVA. All statistical tests were two-tailed, and a p-value of less than 0.05 was considered statistically significant.

## Results

A total of 90 implant-supported restorations placed in the maxillary anterior region were evaluated in this study, with 30 (33.3%) implants included in each implant system group. The demographic and clinical characteristics of the study participants are shown in Table 1. Forty-three (47.8%) patients belonged to the 36-50 years age group, 56 (62.2%) were females, 70 (77.8%) were non-smokers, 58 (64.4%) had a thick gingival biotype, and 54 (60.0%) exhibited a medium smile line. The most frequently restored implant sites were the maxillary central incisors.

**Table 1:** Demographic and clinical characteristics of study participants (n = 90) Data are presented as frequency and percentage.

Variable	Category	Frequency, n (%)
Age (years)	18-35	29 (32.2)
	36-50	43 (47.8)
	51-65	18 (20.0)
Sex	Male	34 (37.8)
	Female	56 (62.2)
Smoking status	Non-smoker	70 (77.8)
	Smoker (<10/day)	13 (14.4)
	Smoker (>10/day)	7 (7.8)
Gingival biotype	Thin (≤1 mm)	32 (35.6)
	Thick (>1 mm)	58 (64.4)
Smile line	Low	18 (20.0)
	Medium	54 (60.0)
	High	18 (20.0)
Implant site	Right canine	11 (12.2)
	Right lateral	16 (17.8)
	Right central	21 (23.3)
	Left central	20 (22.2)
	Left lateral	14 (15.6)
	Left canine	8 (8.9)

The distribution and characteristics of the implant systems are presented in Table 2. Significant differences were observed in the implant diameter and length among the three groups (p < 0.05). Osstem implants had the largest mean implant diameter, while Straumann implants had the largest mean implant length. Internal implant-abutment connections were more commonly observed in Straumann and Nobel Biocare implant systems than in Osstem implants. Significant differences were also noted in the crown material distribution and abutment type among the groups (p < 0.05). However, no statistically significant differences were observed in implant placement timing, loading protocol, crown retention type, or abutment material.

**Table 2:** Distribution of implant systems and their characteristics among the study groups. Continuous variables are presented as mean ± standard deviation, and categorical variables are presented as frequency and percentage. One-way analysis of variance (ANOVA) was used for comparison of implant diameter and implant length among the study groups. Chi-square test was used for categorical variables. *p-Value less than 0.05 was considered statistically significant. SD: standard deviation, CI: confidence interval; χ²: chi-square test, F: ANOVA test statistic.

Implant characteristics		Straumann (n = 30)	Nobel Biocare (n = 30)	Osstem (n = 30)	Test value	p-value
Diameter (mm)	Mean ± SD (CI at 95%)	3.8 ± 0.3 (3.69-3.91)	3.9 ± 0.4 (3.75-4.05)	4.1 ± 0.3 (3.99-4.21)	F = 6.21	0.003*
Length (mm)	Mean ± SD (CI at 95%)	12.5 ± 1.2 (12.05-12.95)	12.0 ± 1.5 (11.44-12.56)	11.5 ± 1.3 (11.01-11.99)	F = 4.87	0.010*
Connection type, n (%)	External	2 (6.7)	1 (3.3)	10 (33.3)	χ² = 15.32	< 0.001*
	Internal	28 (93.3)	29 (96.7)	20 (66.7)		
Placement timing, n (%)	Early	18 (60.0)	15 (50.0)	12 (40.0)	χ² = 2.40	0.301
	Delayed	12 (40.0)	15 (50.0)	18 (60.0)		
Loading protocol, n (%)	Immediate	10 (33.3)	8 (26.7)	6 (20.0)	χ² = 1.40	0.497
	Delayed	20 (66.7)	22 (73.3)	24 (80.0)		
Crown material, n (%)	All-Ceramic (Zirconia)	20 (66.7)	18 (60.0)	12 (40.0)	χ² = 13.75	0.032*
	Lithium disilicate	6 (20.0)	8 (26.7)	10 (33.3)		
	E.max	2 (6.7)	2 (6.7)	4 (13.3)		
	Porcelain fused to metal	2 (6.7)	2 (6.7)	4 (13.3)		
Crown retention, n (%)	Cement-retained	18 (60.0)	20 (66.7)	22 (73.3)	χ² = 1.20	0.549
	Screw-retained	12 (40.0)	10 (33.3)	8 (26.7)		
Abutment type, n (%)	Custom	28 (93.3)	26 (86.7)	20 (66.7)	χ² = 8.30	0.016*
	Prefabricated	2 (6.7)	4 (13.3)	10 (33.3)		
Abutment material, n (%)	Gold alloy	2 (6.7)	4 (13.3)	6 (20.0)	χ² = 7.60	0.107
	Zirconia	22 (73.3)	20 (66.7)	14 (46.7)		
	Titanium	6 (20.0)	6 (20.0)	10 (33.3)		

The esthetic outcomes assessed using PES, WES, and VAS scores are shown in Table 3. Straumann implants demonstrated the highest mean PES score (12.1 ± 1.4), followed by Nobel Biocare (11.8 ± 1.5) and Osstem implants (9.9 ± 1.7), with statistically significant differences among the groups (p < 0.001). Similarly, the WES scores were significantly higher for Straumann and Nobel Biocare implants than for Osstem implants (p < 0.001). A PES score of ≥10 was achieved in 27 (90.0%) Straumann implants, 25 (83.3%) Nobel Biocare implants, and 16 (53.3%) Osstem implants. Similarly, WES scores of ≥8 were observed more frequently in the Straumann and Nobel Biocare implant systems than in the Osstem implants. Mean VAS scores also demonstrated significantly superior esthetic satisfaction for Straumann (88.4 ± 7.2) and Nobel Biocare implants (85.6 ± 8.1) than for Osstem implants (74.3 ± 10.5) (p < 0.001). Overall, the Straumann and Nobel Biocare implant systems exhibited significantly better esthetic outcomes than the Osstem implants in the maxillary anterior region.

**Table 3:** Comparison of esthetic outcomes among different implant systems Data are presented as mean ± standard deviation and frequency with percentage. One-way analysis of variance (ANOVA) was used for comparison of mean PES, WES, and VAS scores, while the chi-square test was used for categorical comparisons, and *p-value < 0.05 was considered statistically significant. PES: Pink Esthetic Score; WES: White Esthetic Score; VAS: Visual Analog Scale; SD: standard deviation; χ²: chi-square test; F: ANOVA test statistic; CI: confidence interval

Aesthetic outcome	Measure	Straumann	Nobel Biocare	Osstem	Test value	p-value
PES	Mean ± SD (CI at 95%)	12.1 ± 1.4 (11.58-12.62)	11.8 ± 1.5 (11.24-12.36)	9.9 ± 1.7 (9.27-10.53)	F = 18.34	< 0.001*
WES	Mean ± SD (CI at 95%)	8.5 ± 0.9 (8.16-8.84)	8.3 ± 1.0 (7.93-8.67)	7.1 ± 1.2 (6.65-7.55)	F = 15.22	< 0.001*
PES ≥ 10	n (%)	27 (90.0%)	25 (83.3%)	16 (53.3%)	χ² = 11.53	0.003*
WES ≥ 8	n (%)	25 (83.3%)	23 (76.7%)	12 (40.0%)	χ² = 14.40	< 0.001*
VAS	Mean ± SD (CI at 95%)	88.4 ± 7.2 (85.71-91.09)	85.6 ± 8.1 (82.58-88.62)	74.3 ± 10.5 (70.38-78.22)	F = 20.97	< 0.001*

Post-hoc analysis revealed no significant differences between Straumann and Nobel Biocare for PES (mean diff. = 0.3, p = 0.426), WES (0.2, p = 0.419), or VAS (2.8, p = 0.162). In contrast, Straumann significantly outperformed Osstem for PES (2.2, p < 0.001), WES (1.4, p < 0.001), and VAS (14.1, p < 0.001). Similarly, Nobel Biocare showed significantly higher scores than Osstem for PES (1.9, p < 0.001), WES (1.2, p < 0.001), and VAS (11.3, p < 0.001). Thus, both Straumann and Nobel Biocare achieved comparable aesthetic outcomes, while Osstem yielded consistently inferior results across all parameters (Table 4).

**Table 4:** Tukey post-hoc analysis with Bonferroni adjustment *p-value < 0.05 was considered statistically significant. CI: confidence interval; PES: Pink Esthetic Score; WES: White Esthetic Score; VAS: Visual Analog Scale

Parameter	Comparison	Mean difference (CI at 95%)	Test value	p‑value
PES	Straumann vs. Nobel Biocare	0.3 (-0.45, +1.05)	t = 0.80	0.426
	Straumann vs. Osstem	2.2 (+1.40, +3.00)	t = 5.47	< 0.001*
	Nobel Biocare vs. Osstem	1.9 (+1.07, +2.73)	t = 4.59	< 0.001*
WES	Straumann vs. Nobel Biocare	0.2 (-0.29, +0.69)	t = 0.81	0.419
	Straumann vs. Osstem	1.4 (+0.85, +1.95)	t = 5.11	< 0.001*
	Nobel Biocare vs. Osstem	1.2 (+0.63, +1.77)	t = 4.21	< 0.001*
VAS	Straumann vs. Nobel Biocare	2.8 (-1.16, +6.76)	t = 1.42	0.162
	Straumann vs. Osstem	14.1 (+9.45, +18.75)	t = 6.07	< 0.001*
	Nobel Biocare vs. Osstem	11.3 (+6.45, +16.15)	t = 4.67	< 0.001*

## Discussion

The present study evaluated the esthetic outcomes of three commonly used implant systems, namely, Straumann, Nobel Biocare, and Osstem implants, in the maxillary anterior region. The findings demonstrated that the Straumann and Nobel Biocare implant systems exhibited significantly superior esthetic outcomes compared with the Osstem implants. These results emphasize the importance of implant design, implant-abutment connection, prosthetic components, and peri-implant soft tissue management in achieving favorable esthetic rehabilitation in the anterior maxilla.

The demographic profile of the present study demonstrated a predominance of female patients and patients belonging to the 36-50 years age group. Similar observations have been reported in a previous esthetic implant study, where middle-aged individuals commonly sought implant-supported rehabilitation because of increasing esthetic awareness and functional concerns [14]. The majority of patients in the present study exhibited a thick gingival biotype and medium smile line, which are considered favorable factors for achieving stable peri-implant soft tissue contours and esthetic outcomes. Thick gingival biotypes are generally associated with reduced soft-tissue recession and improved masking of implant restorative components [15].

The present study demonstrated significantly higher PES and WES scores for the Straumann and Nobel Biocare implant systems than for the Osstem implants. The superior esthetic performance observed with Straumann and Nobel Biocare implants may be attributed to several factors, including implant macrodesign, implant-abutment precision fit, platform switching concepts, and the greater use of custom abutments and all-ceramic restorations in these groups. Internal implant-abutment connections, which are more commonly observed in Straumann and Nobel Biocare implants, have been associated with reduced crestal bone loss and improved peri-implant soft tissue stability [6]. Laurell and Lundgren [16] reported that premium implant systems demonstrated favorable maintenance of marginal bone levels, which may contribute to enhanced peri-implant soft tissue stability and improved esthetic outcomes over time.

The findings of the present study are consistent with those of Barwacz et al. [9], who reported favorable esthetic outcomes around implant-abutment configurations with stable peri-implant soft tissue conditions and high PES scores. Similarly, Belser et al. [12] demonstrated that implants restored with optimized prosthetic protocols and favorable soft tissue management could achieve high PES and WES values in the anterior region of the maxilla. The present study also supports previous evidence suggesting that premium implant systems with advanced surface characteristics and prosthetic precision may provide superior esthetic outcomes.

Higher WES scores observed in the Straumann and Nobel Biocare implant systems may also be related to the greater use of zirconia-based all-ceramic restorations and custom abutments. Zirconia abutments and ceramic restorations have been shown to improve light transmission, translucency, and color matching in esthetically demanding areas [17]. In contrast, the greater proportion of metal-based restorative components and prefabricated abutments in the Osstem group may have negatively influenced the soft tissue appearance and final prosthetic esthetics [18].

The VAS scores in the present study also demonstrated significantly greater esthetic satisfaction in the Straumann and Nobel Biocare groups than in the Osstem group. In the present study, implant systems that demonstrated superior clinician-reported esthetic outcomes using PES and WES also exhibited higher VAS satisfaction scores. However, recent evidence suggests that clinician-based esthetic assessments may not always correlate with patients’ perceptions of esthetic success. Sadilina et al. [19], in a systematic review and individual participant data meta-analysis, reported no significant association between PES/WES scores and patient-reported esthetic satisfaction following implant-supported restorations in the esthetic zone. Differences in patient expectations, psychological perceptions, smile characteristics, and subjective esthetic preferences may contribute to this discrepancy. Nevertheless, the higher VAS scores observed in the present study may indicate that improved peri-implant soft tissue and prosthetic esthetics positively influenced overall patient satisfaction.

Another important observation of the present study was the high prevalence of internal implant-abutment connections in the Straumann and Nobel Biocare systems. Internal connections are associated with improved force distribution, enhanced microbial sealing, and better preservation of peri-implant hard and soft tissues. These biomechanical and biological advantages may explain the improved esthetic outcomes observed in the present study [20].

The present study also highlights the significance of prosthetic rehabilitation protocols in determining the final esthetic success. Appropriate abutment selection, emergence profile development, crown contouring, and occlusal harmony are essential for maintaining peri-implant soft tissue stability and natural esthetics [4,5]. The use of custom abutments in most Straumann and Nobel Biocare cases may have contributed to improved adaptation of the peri-implant soft tissues and more favorable emergence profiles.

From a clinical perspective, the findings of the present study suggest that implant system selection plays a significant role in achieving predictable esthetic outcomes in the maxillary anterior region. Implant systems with internal connections, advanced prosthetic components, and greater restorative flexibility may provide superior esthetic integration and patient satisfaction. The use of custom abutments and all-ceramic restorations should be considered whenever esthetic demands are high. Careful treatment planning, soft tissue evaluation, and prosthetic execution are critical for successful implant-supported rehabilitation in the esthetic zone.

Despite these significant findings, the present study had certain limitations. Although the study was prospectively conducted, its observational, non-randomized design may have introduced selection bias and residual confounding. The study was conducted at a single institutional center with a relatively limited sample size, which may have affected the generalizability of the findings. Furthermore, treatment-related variables, including implant dimensions, implant-abutment connection design, crown material, abutment type, retention type, loading protocol, and gingival phenotype, were not standardized or controlled because treatment decisions were based on routine clinical practice. These factors may have independently influenced the observed esthetic outcomes, and no multivariable adjustment was performed to account for their potential confounding effects. In addition, the six-month follow-up period may not fully reflect long-term peri-implant soft tissue maturation, esthetic stability, and patient-reported outcomes. Therefore, the findings should be interpreted as demonstrating clinical associations rather than causal effects. Future multicenter prospective studies with larger sample sizes, standardized treatment protocols, longer follow-up periods, and multivariable analyses are recommended to further validate these findings.

## Conclusions

Within the limitations of the present study, Straumann and Nobel Biocare implant system groups were associated with more favorable esthetic outcomes than the Osstem implant group in the maxillary anterior region, as reflected by higher PES, WES, and VAS scores. These observed differences may have been influenced not only by implant system characteristics but also by variations in implant-abutment connection design, prosthetic components, and other treatment-related factors inherent to routine clinical practice. Therefore, the findings should be interpreted as demonstrating clinical associations rather than causal effects. Careful implant selection, soft tissue management, and prosthetic rehabilitation remain essential for achieving predictable esthetic outcomes and patient satisfaction in implant-supported restorations within the esthetic zone.

## References

[1] Guillaume B (2016). Dental implants: a review. Morphologie.

[2] Shyamkumar NT, Patil R, Bhukal S, Mukhopadhyay N, Bhardwaj T, Gupta S (2026). Behavioral determinants of patients' willingness to undergo dental implant therapy: a health belief model-based cross-sectional study. Cureus.

[3] Cho SC, Froum SJ, Kamer AR, Loomer PM, Romanos G, Demiralp B (2015). Implants in the anterior maxilla: aesthetic challenges. Int J Dent.

[4] Nam J, Aranyarachkul P (2015). Achieving the optimal peri-implant soft tissue profile by the selective pressure method via provisional restorations in the esthetic zone. J Esthet Restor Dent.

[5] Dilip Taide P, Faizan M, Salunkhe G, Sutradhar W, Yerra S, Gavara SG, Sharma M (2025). Automated prediction of dental implant success using a mask region-based convolutional neural network on preoperative cone-beam computed tomography scans. Cureus.

[6] Shenoy A, Rohinikumar S, Maiti S, Sivaswamy V, Rajaraman V (2023). Evaluation of peri-implant crestal bone loss with different implant systems, primary stability, bone density and soft tissue thickness: a retrospective study. J Long Term Eff Med Implants.

[7] Parvini P, Trimpou G, Begic A (2023). Esthetic and clinical outcomes after immediate placement and restoration: comparison of two implant systems in the anterior maxilla-a cross-sectional study. Clin Implant Dent Relat Res.

[8] (2013). World Medical Association Declaration of Helsinki: ethical principles for medical research involving human subjects. JAMA.

[9] Barwacz CA, Stanford CM, Diehl UA, Cooper LF, Feine J, McGuire M, Scheyer ET (2018). Pink Esthetic Score outcomes around three implant-abutment configurations: 3-year results. Int J Oral Maxillofac Implants.

[10] Kanout C (2023). Evaluation of the translucency properties for CAD/CAM full ceramic crowns fabricated from glass ceramics (E.max) or high translucency zirconia (Lava Plus): a clinical study. Cureus.

[11] Fürhauser R, Florescu D, Benesch T, Haas R, Mailath G, Watzek G (2005). Evaluation of soft tissue around single-tooth implant crowns: the pink esthetic score. Clin Oral Implants Res.

[12] Belser UC, Grütter L, Vailati F, Bornstein MM, Weber HP, Buser D (2009). Outcome evaluation of early placed maxillary anterior single-tooth implants using objective esthetic criteria: a cross-sectional, retrospective study in 45 patients with a 2- to 4-year follow-up using pink and white esthetic scores. J Periodontol.

[13] Chander NG (2019). Visual analog scale in prosthodontics. J Indian Prosthodont Soc.

[14] Bhagawati SB, Jain SR, Debnath P, Riyaz K, Patil R, Ansari J (2024). Patients' perceptions regarding acceptance of dental implants as an option for the replacement of missing teeth: an observational study. Cureus.

[15] Lee A, Fu JH, Wang HL (2011). Soft tissue biotype affects implant success. Implant Dent.

[16] Laurell L, Lundgren D (2011). Marginal bone level changes at dental implants after 5 years in function: a meta-analysis. Clin Implant Dent Relat Res.

[17] Zembic A, Sailer I, Jung RE, Hämmerle CH (2009). Randomized-controlled clinical trial of customized zirconia and titanium implant abutments for single-tooth implants in canine and posterior regions: 3-year results. Clin Oral Implants Res.

[18] Fenner N, Hämmerle CH, Sailer I, Jung RE (2016). Long-term clinical, technical, and esthetic outcomes of all-ceramic vs. titanium abutments on implant supporting single-tooth reconstructions after at least 5 years. Clin Oral Implants Res.

[19] Sadilina S, Müller NPA, Strauss FJ, Jung RE, Thoma DS, Bienz SP (2025). Patient-reported and clinician-reported esthetic outcomes at implant sites are not associated: a systematic review with individual participant data meta-analysis. Clin Oral Implants Res.

[20] Choi S, Kang YS, Yeo I-SL (2023). Influence of implant-abutment connection biomechanics on biological response: a literature review on interfaces between implants and abutments of titanium and zirconia. Prosthesis.

